# A Case of Pigmented Macules Occurring After Ocular Myasthenia Gravis

**DOI:** 10.7759/cureus.34823

**Published:** 2023-02-09

**Authors:** Kayleigh Huimin Ho, Khang Ning Loo

**Affiliations:** 1 General Medicine, Sengkang General Hospital, Singapore, SGP

**Keywords:** pigmented skin lesion, ashy dermatosis, ocular myasthenia gravis, lichen planus pigmentosus, lichen planus pigmentosus-inversus

## Abstract

Lichen planus pigmentosus inversus (LPP-I) is characterized by the presence of hyperpigmented or hypopigmented lesions on the flexural surfaces of the body. It is a rare variant of lichen planus pigmentosus with unknown etiology. We report a case of a male presented with LPP-I three months after diagnosis of ocular myasthenia gravis, highlighting the possible autoimmune association.

## Introduction

Lichen planus pigmentosus-inversus (LPP-I) is a rare variant of lichen planus, a chronic autoimmune skin condition characterized by the development of itchy, flat-topped, purple or brownish-black papules or plaques on the skin. LPP-I is characterized by the presence of hyperpigmented or hypopigmented lesions on the flexural surfaces of the body, such as the axillae, groins, and nape of the neck. The cause of LPP-I is unknown, but it is thought to be an autoimmune disorder that may be triggered by certain medications, infections, or other underlying medical conditions. The condition is more common in women and in people of Asian descent.

## Case presentation

A 67-year-old Indian male presented with an itchy, non-photo-distributed rash on the lower back for 10 days. He also noticed pigmented rash in both his axillae and the left inframammary area of his chest. He had no prior history of skin rash or drug allergies. His medical history included hypertension for the past eight years, which was treated with telmisartan.

Three months prior to his current presentation, his ophthalmologist noted that he had left head tilt associated with right eye hypertropia on the straightening of his head. His eye examination revealed fatigable ptosis of both eyelids with a positive Cogan’s lid twitch sign. Single fiber electromyography showed mild to moderate neuromuscular transmission defect but no significant decremental response seen in repetitive nerve stimulation. Anti-acetylcholine receptor antibody was negative. Magnetic resonance imaging of his brain and orbits were normal. Computed tomography of his chest did not show any thymic mass. He was diagnosed to have ocular myasthenia gravis and was commenced on pyridostigmine. His ptosis and head tilt showed improvement with pyridostigmine on subsequent follow-up. 

**Figure 1:**
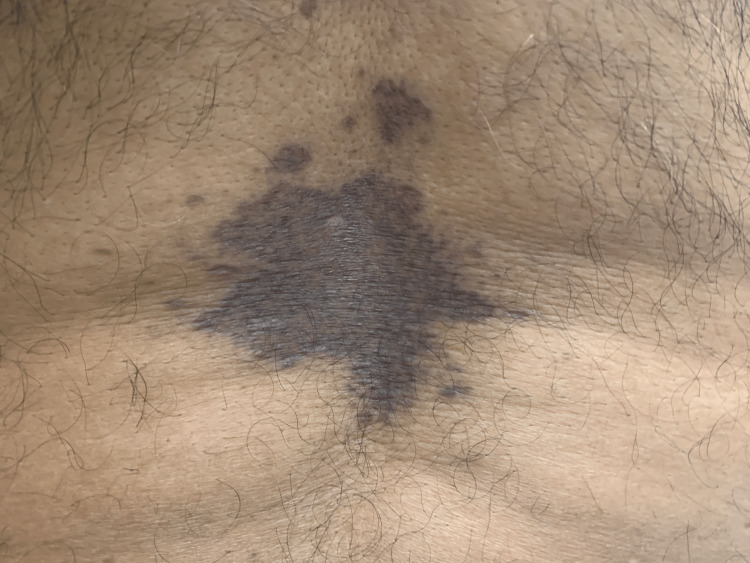
Hyperpigmented lesion at the back.

**Figure 2:**
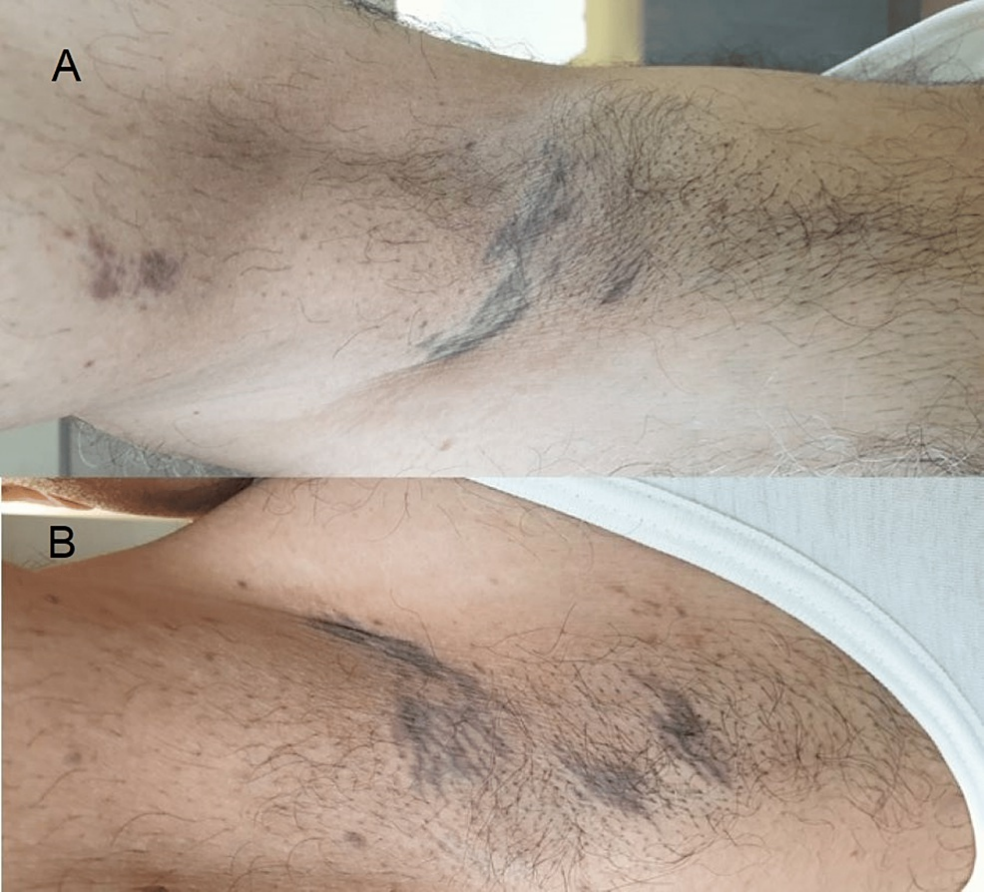
Hyperpigmented linear streaks on (A) left axilla, and (B) right axilla

**Figure 3:**
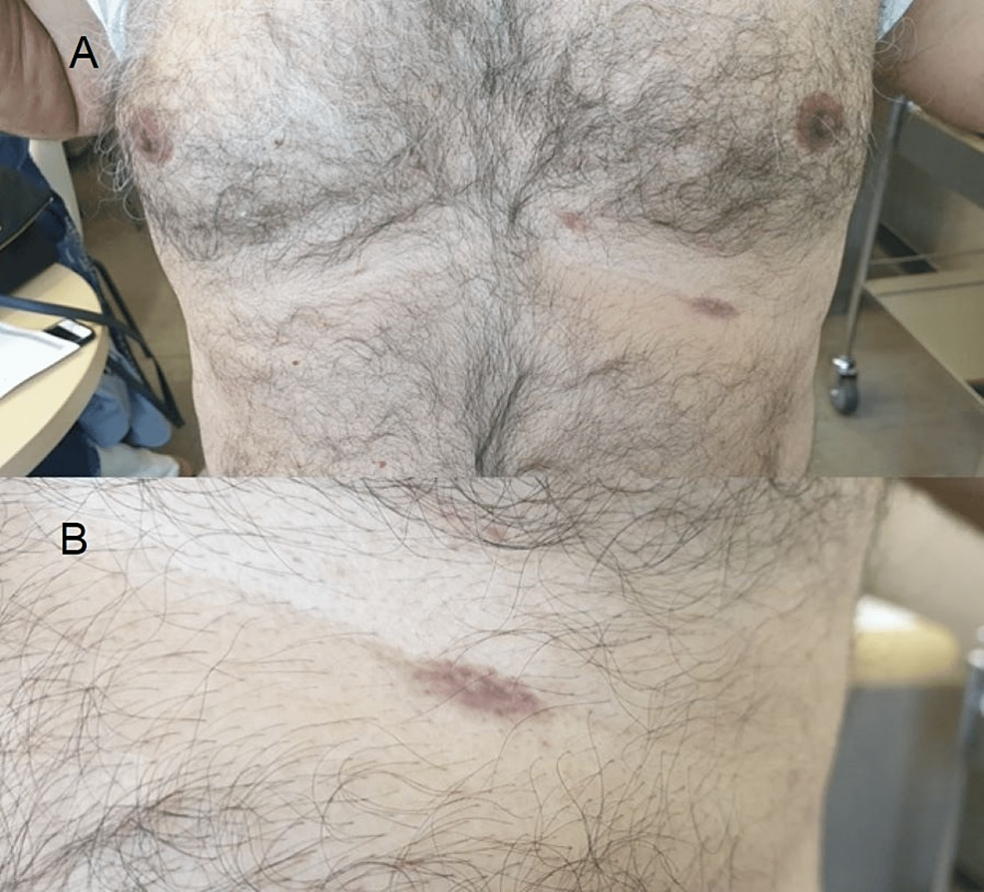
(A) Two erythematous macules on the left chest at the inframammary line. (B) Closer view of one of the macules.

A dermatological examination showed a 5 cm x 5 cm hyperpigmented, purplish patch with an irregular border at the center of his back (Figure 1). Several hyperpigmented linear streaks were noted in both axillae (Figure 2). Two erythematous macules were noted on his chest at the left inframammary region (Figure 3). There was no skin lesion on the sun-exposed area or other flexural areas.

**Figure 4:**
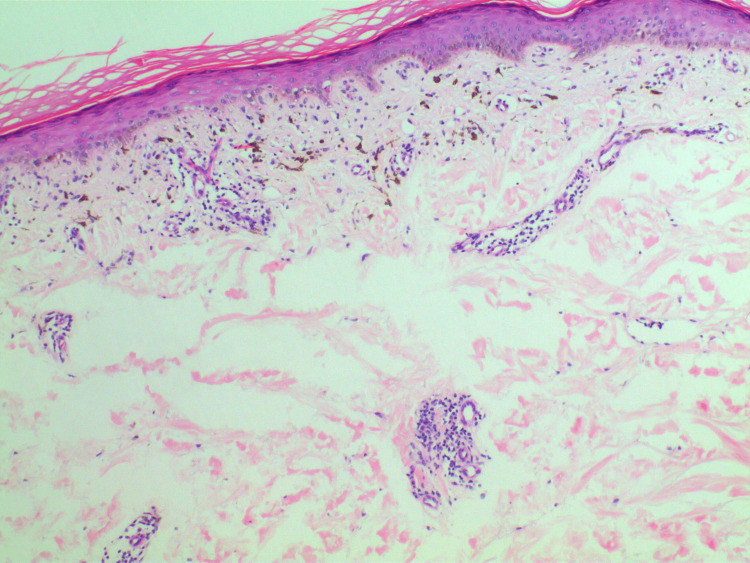
Hematoxylin and Eosin (H&E) x100 magnification. The skin biopsy shows vacuolar interface reaction with pigmentary incontinence.

Full blood count, and renal, liver, and thyroid function tests were normal. Screening for diabetes was normal. Hepatitis B surface antigen and hepatitis C viral serology were negative. A 4 mm punch biopsy was performed on his back lesion. Microscopically, the epidermis showed mild basal vacuolation with rare apoptotic keratinocytes. There was pigmentary incontinence and a mild superficial perivascular infiltrate of lymphocytes (Figure 4).

Essentially, our patient developed several hyperpigmented macules of varying size at several flexural areas of the trunk which were non-sun exposed. Given his South Asian lineage and correlation with histopathological findings, a lichen planus pigmentosus inversus diagnosis was made. 

## Discussion

Lichen planus pigmentosus (LPP) is first described by Bhutani et al. in 1974 [1]. In their case series of 40 Indian patients, the lesions usually start as small, ill-defined, oval-to-round macules that evolve into confluent, uniform sheets of pigmented areas. The color varies among patients from slate-blue to steel-grey. Distribution is variable, commonly involving the face, upper extremities, abdomen, and upper back. Less commonly it can also involve axillae, groin, inframammary area, and bald scalp. The lesions are often bilaterally symmetrical, with predominance in the sun-exposed area [1]. The disease is more commonly seen in Indians although it has also been described in other ethnicities. When the disease affects non-sun exposed, intertriginous and flexural areas, it is known as lichen planus pigmentosus inversus, a variant of LPP [2]. It predominantly affects women in the menopausal or perimenopausal stage [3]. While the cause and the mechanism remain unclear, the triggers have been attributed to friction and tight clothing. 

Both lichen planus pigmentosus and lichen planus share similar immunopathogenesis, with an altered cellular immune response mediated by T lymphocytes. CD8+ T lymphocytes recognize and attack epidermal keratinocytes which leads to intense pigmentary incontinence in LPP [4]. LPP-I has a significant association with a metabolic disease with diabetes mellitus or hypertension [5]. The association with hepatitis C and better response to tacrolimus over steroids support the immune-mediated correlation [6]. Despite the name, most patients with LPP do not develop lichen planus elsewhere in the body. Some cases of LPP were associated with autoimmune diseases. One large case series of LPP by Vinay et al. reported 13% of the patients had a history of autoimmune conditions, whereas 14% had existing atopic diseases during their eight-year study period [7]. Our patient has ocular myasthenia gravis without evidence of autoantibodies and thymoma. Although LPP-I can be associated with autoimmune diseases, there is no reported case of its association with myasthenia gravis in our literature search.

The disease is benign and generally has a longer course than other lichen planus variants and may respond well with topical corticosteroids and occasionally require tacrolimus ointment for resolution. Phototherapy is not recommended for the risk of pigmentation. Our patient was started on topical betamethasone valerate 0.05% cream for 6 weeks. Subsequent visits did not show any new lesions or progression and the pigmentation remained stable.

## Conclusions

In conclusion, LPP-I is a rare variant of lichen planus characterized by the presence of hyperpigmented lesions on the flexural surfaces of the body. LPP-I has to be distinguished from post-inflammatory hyperpigmentation due to conditions such as dermatitis, lichenoid drug eruption, or other aetiologies, as the management will then be tailored to specific underlying causes. In our patient, the onset of ocular myasthenia gravis preceding the skin lesions could point to a possible autoimmune association, which has not previously been reported in the literature. Treatment options include topical corticosteroids as the first line, with calcineurin inhibitors as an alternative in refractory cases. The disease is self-limiting and generally has a good prognosis.
